# Accidental Choking in Children: An Area To Be Focused on

**DOI:** 10.7759/cureus.22459

**Published:** 2022-02-21

**Authors:** Uruthirapasupathi Mayorathan, Sriluxayini Manikkavasakar, Sellaiah Pranavan

**Affiliations:** 1 Forensic Medicine, Jaffna Teaching Hospital, Jaffna, LKA; 2 Radiology, Jaffna Teaching Hospital, Jaffna, LKA

**Keywords:** prevention, airway obstruction, foreign body, asphyxia, choking

## Abstract

Choking is one of the important modes of death in young children who are dying of unintentional injuries. Anatomical and physiological characteristics while eating could increase the incidence of choking in children under four years. Here, we have described two classical cases of choking in young children and discussed the important areas to be addressed. Parental awareness and education will be the important strategy that can prevent the incidence of choking.

## Introduction

Choking is defined as mechanical obstruction of the internal airway, and the presence of a foreign body results in impaired respiratory function [1]. Choking is a significant cause of death due to unintentional injuries in young children but has been observed up to the age of 14 years, and some studies have stated that around 60-80% of deaths due to choking are related to food [2]. Anatomical and physiological characteristics that are peculiar to children may be a reason for increased choking incidents among young children while eating [1]. Further, strategies developed based on knowledge, and legal requirements to prevent nonfood-related choking (e.g., small toys) have resulted in a massive reduction in morbidity and mortality due to this type of injury [3]. Nevertheless, as the prevalence of food-related choking appears to be higher than previously reported [3], the value of preventive actions should be emphasized to parents, guardians, and caregivers [4].

## Case presentation

Case 1

**Figure 1:**
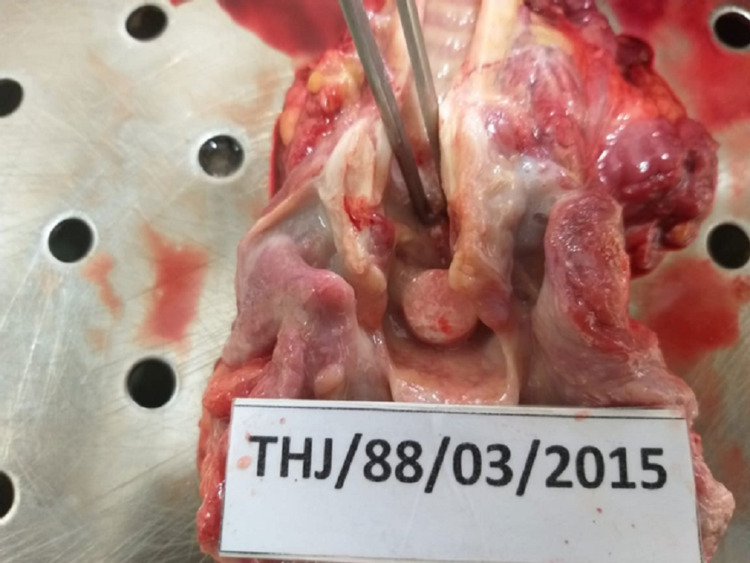
A paracetamol tablet was struck at the epiglottic region

A previously healthy three-year-old child was playing in her grandfather’s room. The grandfather had left the room for a while, during that time the child developed noisy breathing and restlessness. She became cyanotic, was aggressive, and clutched her neck with both hands. Her parents rushed her to the nearby divisional hospital, but she was pronounced dead at the outpatient department. An autopsy was performed at the Jaffna Teaching hospital (TH), Jaffna, Sri Lanka, which revealed that airway obstruction was due to a paracetamol tablet lodged in the epiglottic region (Figure 1).

Case 2

**Figure 2:**
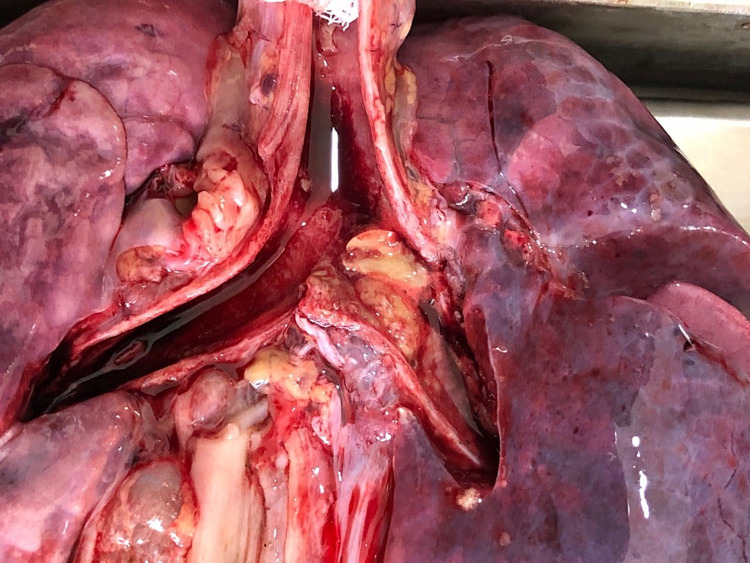
Obstruction by two halves of peanuts at the level of bifurcation

A previously healthy 2.5-year-old female child was brought to TH Jaffna emergency department after suddenly collapsing while she was being fed by her father, who was under the influence of alcohol. She was admitted to the accident and emergency department motionless. The child could not be revived despite optimal care. The autopsy revealed the presence of two half-peanuts at the bifurcation of the trachea and some food particles in the lower airways (Figure 2).

## Discussion

Choking is a type of asphyxia where internal air passages, including the pharynx, hypopharynx, and trachea, are obstructed by solid foreign bodies [5]. Choking is predominantly accidental; however, it may rarely be homicidal if a gag placed in the mouth slides down into the pharynx after being soaked with saliva [5].

Children under four years of age and those suffering from chewing and swallowing difficulties are more vulnerable to choking [1]. Food-related choking in children occurs due to children younger than two years of age not developing molar teeth. Thus, while children below two years of age bite the food with their incisors, they cannot grind it for smooth swallowing. Further, even though children aged three to four years show eruption of molar teeth, their chewing practices remain insufficient, especially with inappropriate foods for their age [6]. Additionally, children at this age get easily distracted and may not pay adequate attention while eating.

Neurological disorders, neuromuscular pathologies, developmental delay, and traumatic and non-traumatic brain conditions considerably contribute to disturbances in swallowing, apart from certain primary and secondary medical conditions. A child’s habits and behaviors, such as walking, running, talking, laughing, and urgency in eating, could add to this risk of choking. Furthermore, certain games played by children could lead to fatal choking, e.g., throwing nuts or candies into the air and trying to catch them by the mouth, and stuffing foods into their mouth, among others [1]. A common cause for choking is food, i.e., meatballs, sausage, popcorn, candy, bone pieces, and pieces of dog food. Other objects that obstruct airways include buttons, toys with small parts, toys that can fit in a child’s mouth, small balls, marbles, balloons, small hair ties, rubber bands, pen caps, small button-type batteries, and refrigerator magnets.

Chocking can kill an individual within minutes, and quick intervention is needed to save the victim's life. First, ask the child to speak or cough to check for choking due to an object. If he/she can talk effectively and cough, there is no need for an intervention, and the patient is encouraged to cough to get rid of the obstruction. If the child can’t cough or speak or develops stridor or cyanosis, a series of blows on the back and manual abdominal thrusts may be needed to expel the object. While the blows and manual thrusts can be performed separately, a combination yields better results than individually [7]. Typically, hospitalized victims following suspected choking usually undergo imaging, which can visualize foreign bodies, hyperinflated lungs, either unilateral or bilateral, collapsed lung, or a midline shift; however, radiographs with non-significant findings cannot be used to exclude choking [8]. Rigid bronchoscopy is the strategy of choice for diagnosis as well as management of choking and when performed as early as possible; it has a success rate of 95% and a low rate of procedure-related complications (<1%) [8]. Even though flexible bronchoscopy is frequently used in hospitals because it is safe and can be conveniently used to diagnose and remove foreign bodies, the American Thoracic Society recommends a rigid bronchoscope for foreign body removal [8].

An autopsy can reveal both typical signs of asphyxia and the objects that caused the death in deceased individuals. Signs of asphyxia are cyanosis, congestion of the eye and other organs, pulmonary edema, and petechial hemorrhages over the face, eyelids, behind the ears, submucosal, subserosal, and subcapsular surfaces of internal organs, and interlobar fissures (Tardieu’s spots) [9].

Prevention of choking requires close monitoring, proper labeling of food and non-food items with warnings of choking hazard, and, if necessary, recalling the product from the market [1]. The most important preventive strategy is educating parents and caregivers [4]; however, large online markets and reselling of recalled goods have created serious threats as these products do not carry proper labeling.

## Conclusions

Both cases described here represent classic examples of choking that occurred in children in vulnerable age groups. These cases highlight the importance of parental awareness and education, including the first aid measures, which can prevent such tragedies. In addition to that, the establishment of a nationwide food-related choking incidence surveillance and reporting system could be helpful to develop prevention methods in the future.

## References

[1] (2010). Prevention of choking among children. Pediatrics.

[2] Lorenzoni G, Azzolina D, Baldas S (2019). Increasing awareness of food-choking and nutrition in children through education of caregivers: the CHOP community intervention trial study protocol. BMC Public Health.

[3] Lorenzoni G, Lanera C, Azzolina D, Baldas S, Messi G, Gregori D (2021). Assessing school-based intervention strategies to foster the prevention of choking injuries in children: The results of the CHOP (CHOking Prevention) trial. Health Soc Care Community.

[4] Takamiya M, Niitsu H, Saigusa K, Dewa K (2016). Pediatric autopsy case of asphyxia due to salmon egg (ikura) aspiration. Pediatr Int.

[5] Dolinak D, Matshes E, Lew E (2005). Forensic Pathology: Principles, and Practice. First Edition. California, USA: ELSEVIER ACADEMIC PRESS.

[6] Reilly JS, Cook SP, Stool D, Rider G (1996). Prevention and management of aerodigestive foreign body injuries in childhood. Pediatr Clin North Am.

[7] Sumner SM, Grau PE (1982). Emergency! First aid for choking. Nursing.

[8] Mandal A, Kabra SK, Lodha R (2015). Upper Airway Obstruction in Children. Indian J Pediatr.

[9] de Alwis LB (2007). Medico-Legal Aspects of Injuries. First Edition.

